# *In Vitro* Analysis of Photosensitizer Accumulation for Assessment of Applicability of Fluorescence Diagnosis of Squamous Cell Carcinoma of Epidermolysis Bullosa Patients

**DOI:** 10.1155/2013/521281

**Published:** 2012-12-30

**Authors:** Patrick Larisch, Thomas Verwanger, Kamil Onder, Barbara Krammer

**Affiliations:** ^1^Division of Molecular Tumorbiology, Department of Molecular Biology, University of Salzburg, Hellbrunnerstrasse 34, 5020 Salzburg, Austria; ^2^Division of Molecular Dermatology, Department of Dermatology, Paracelsus Medical University, Muellner Hauptstrasse 48, 5020 Salzburg, Austria

## Abstract

Epidermolysis bullosa (EB) is a group of inherited skin disorders characterized by blistering following mechanical trauma. Chronic wounds of EB patients often lead to tumors such as squamous cell carcinoma (SCC). Early diagnosis may prevent its invasive growth—frequently the reason of premature mortality of EB-patients. Early detection of tumors is achieved by fluorescence diagnosis (FD), where photosensitizers localize selectively in tumors and fluoresce upon illumination. Excessive accumulation of photosensitizers in inflamed areas, as occasionally found at chronic wounds and tumors due to inflammatory processes, leads to false-positive results in FD. This study analyzed accumulation kinetics of the photosensitizers hypericin and endogenous protoporphyrin IX (PpIX) in different skin cell lines including the three EB subtypes under normal and proinflammatory conditions (stimulated with TNF-alpha). The aim was to assess the applicability of FD of SCC in EB. All cell lines accumulate hypericin or PpIX mostly increasing with incubation time, but with different kinetics. SCC cells of recessive dystrophic EB (RDEB) accumulate less hypericin or PpIX than nonmalignant RDEB cells. Nevertheless, tumor selectivity *in vivo* might be existent. Non-EB cell lines are more active concerning photosensitizer enrichment. Proinflammatory conditions of skin cell lines seem to have no major influence on photosensitizer accumulation.

## 1. Introduction

Epidermolysis bullosa (EB) is a group of skin disorders which are genetically determined. They are characterized by blistering of the skin and mucosa following mechanical trauma [1–3]. EB can be divided into three classes. 

EB simplex (EBS) is the most common form of EB. Its inheritance is normally autosomal dominant but in some cases an autosomal recessive trait can be found. The blister formation begins intraepidermally with a subnuclear disruption of the basal keratinocytes. The reason for this is mutations in specific genes encoding for keratin 5 and keratin 14 (KRT5 and KRT14) [4, 5] and for plectin (PLEC1) [6]. 

EB junctionalis (EBJ) is a group of autosomal recessive disorders. There are two main categories within this group of EB, the Herlitz (lethal) and non-Herlitz (nonlethal) form. The tissue separation of these forms is through the lamina lucida of the basement-membrane zone beneath the plasma membrane of epidermal basal cells. Nonscarring blistering is the result of this separation. Mutations in genes encoding for laminin 5 subunits (LAMA3, LAMC2, and LAMB3) and collagen, type XVII, alpha 1 (COL17A1) are causative for this form of EB [7].

EB dystrophica (EBD) has an autosomal recessive or dominant inheritance. The blistering level of this type of EB lies below the lamina densa of the epidermal basement membrane. Mutations are occurring in COL7A1, the gene encoding for collagen, type VII, alpha 1 [8].

All these forms of EB are resulting in the pain of blistering, inflammation, and in some cases scarring and cancer because of loss of the skin's barrier function [9].

The chronic wounds of EB patients are accompanied by inflammatory processes, which may promote induction and growth of skin tumors such as squamous cell carcinoma (SCC), especially when the inflammation lasts for a long period or is derailed [10]. Early diagnosis of SCC is important, since early stages of SCC can be treated more easily than invasively growing SCC, which often is the main reason of premature mortality of the EB patients. To this purpose, a new, effective, and noninvasive technique for early detection of SCC would be offered by fluorescence diagnosis (FD) using a photosensitizer. The latter localizes selectively in tumor tissue and is able to fluoresce upon irradiation with visible light of a wavelength matching the absorption spectrum of the substance. This modality can be applied for tumor diagnosis, even in early stages, and it is especially helpful in fluorescence-guided resection [11].

Beyond diagnosis, the tumor-localizing photosensitizer is able to kill the target cells when light activated. In the presence of oxygen, most photosensitizers generate either superoxide radicals, that might form peroxides and hydroxyl radicals in a type I reaction, or singlet oxygen molecules (^1^O_2_) in a type II reaction. The tumor destruction occurs finally due to reactive oxygen species (ROS) [12] or reactive nitrogen species [13]. This treatment is called “photodynamic therapy (PDT)” and was already used for basal cell carcinoma treatment of an RDEB-patient [14].

Chronic wounds, especially a problem for EB patients, as well as tumors are often accompanied by inflammatory processes, which may lead to false-positive results in FD, decreasing the specificity. The reason for this is unclear, but some clinical studies supposed local immune cells such as macrophages, which invade inflamed areas, as source for an excessive accumulation of the photosensitizer [15–18]. Nevertheless, it cannot be excluded that nonimmune cells accumulate the photosensitizer at a higher rate under inflammatory conditions, and that proinflammatory cytokines could play a role in this process.

Proinflammatory cytokines control inflammation and modulate neovascularisation, cell proliferation, and migration [19]. Inflammation is an essential part of wound healing, but it can turn to a problem, when this controlled process is switching to an uncontrolled or excessive one. This is often seen in diseases like chronic wounds, tumor metastasis, psoriasis, and arthritis [20]. Most of all deregulated wound healing is caused by an increase of interleukin 1 (IL-1alpha and IL-1beta) and tumor necrosis factor-alpha (TNF-alpha) levels [21–24]. Interleukin 6 (IL-6) also seems to play an important role in the pathogenesis of inflammation [25]. On the other hand, secretion of these cytokines was found to be upregulated by PDT [26–28].

As indicated before, detection of early stages of SCC in EB-patients via fluorescence diagnosis would be a new approach to prevent invasive SCC growth by early intervention. Therefore, the aim of the present study should be the analysis of the fluorescence kinetics of photosensitizers in EB cell lines to assess the applicability of FD on SCCs of EB-patients. 

The uptake of an externally applicable photosensitizer such as hypericin and accumulation of the endogenously formed photosensitizer protoporphyrin IX (PpIX) should be analysed in the malignant EB-cell line SCCRDEB4 and compared to nonmalignant EB cell lines and a malignant non-EB cell line. To induce endogenous PpIX formation, its precursor ALA (5-aminolevulinic acid) in the heme biosynthesis will be applied. PpIX is currently successfully used in tumor diagnosis [11]. Hypericin is a plant constituent from St. John's wort with excellent fluorescent properties that can modulate several signaling pathways [29, 30].

To prove the hypothesis that inflammation of tissues causes excessive accumulation of photosensitizers often leading to false-positive results in FD, the effect of proinflammatory conditions on uptake or accumulation of hypericin or PpIX, respectively, in normal and malignant EB-cell lines and their respective reference cell lines should also be analysed. The question here is whether FD is influenced by the proinflammatory state of EB cells.

In order to address these issues, we performed analysis of uptake kinetics on seven different skin cell lines (GABEB, EBS-MD, RDEB-CL, SCCRDEB4 as EB cell lines, representative of the three main subtypes, and HaCat, Skin, A431 as keratinocyte, fibroblast, and SCC cancer cell lines as control and reference). For establishment of a proinflammatory milieu, we stimulated the cells with TNF-alpha (tumor necrosis factor-alpha) to activate proinflammatory pathways [31].

## 2. Material and Methods

### 2.1. Cell Lines

GABEB and EBS-MD (immortalized fibroblasts) cells were obtained from Dr. Johann Bauer (Division of Molecular Dermatology and EB House Austria, Department of Dermatology, Paracelsus Medical University, Salzburg, Austria). RDEB, exactly RDEB-CL cells, were obtained from Professor Guerrino Meneguzzi, Nice, France, and SCCRDEB4 [32] cells were kindly provided by Dr. Andrew South, Dundee, UK. Control cell lines (HaCat, Skin, and A431) were available in our laboratory. For cell lines, EB subtypes and mutations see Table 1.

The SCCRDEB4 cell line was routinely grown in Keratinocyte-SFM (Life Technologies, Vienna, Austria) and RDEB-CL and GABEB cell lines in Keratinocyte-SFM with 100 U/mL penicillin and 0.1 mg/mL streptomycin. Keratinocyte-SFM was always supplemented with bovine pituitary extract and recombinant epidermal growth factor. EBS-MD, Skin, HaCat, and A431 cell lines were cultivated in Dulbecco's modified Eagle's medium (DMEM) containing 4.5 g/L glucose supplemented with 10 mM HEPES, 4 mM l-glutamine, 1 mM Na-pyruvate, 100 U/mL penicillin, 0.1 mg/mL streptomycin, and 10% (v/v) or 5% (v/v) for A431, respectively, foetal bovine serum (FBS) (all from PAA-Laboratories, Linz, Austria or LONZA, Basel, Switzerland) in a humidified atmosphere at 37°C and 5% CO_2_.

### 2.2. Photosensitizers

In preliminary experiments, cell proliferation characteristics were analysed by the MTT assay in order to determine the respective cell number for each cell line, which should be used to yield comparable results due to comparable cell mass and monolayer density. Twenty-four hours after seeding the cells to confluency of about 80% cell culture medium was replaced with medium containing hypericin or the PpIX precursor ALA.

Hypericin is a secondary metabolite predominantly extracted from the *Hypericum perforatum *(St. John's wort). It was purchased from Planta Natural Products (Vienna, Austria) and added to the serum-free medium of the cell cultures in final concentrations of 3 and 5 *μ*M. These concentrations were chosen according to previous work [33]: irradiation of A431, HaCat, and SCCRDEB4 cells with a diagnostic protocol using 3 *μ*M hypericin was sublethal, but using 5 *μ*M hypericin was phototoxic with 40% to 70% survival having therapeutical impact.

Endogenous PpIX is the last molecule in the heme biosynthesis prior to heme and depends on its precursor 5-aminolevulinic acid (ALA). If ALA is given in excess, PpIX is accumulated in cells and can be used as a very effective photosensitizer for many hours. ALA was purchased from Sigma-Aldrich (Vienna, Austria). It was applied to the serum-free medium of the cell cultures in final concentrations of 0.5 and 1 mM. Also, these concentrations were chosen according to a previous study [34], in which 0.5 mM and 1 mM ALA was used to induce PpIX efficiently in a linear relationship for fluorescence detection of mononuclear and circulating tumor cells.

Handling with photosensitizers was performed under subdued light conditions.

### 2.3. Induction of Proinflammatory Milieu

Fibroblast and keratinocyte cell lines were incubated with lipopolysaccharide (LPS) or TNF-alpha (both Sigma-Aldrich, Vienna, Austria) in preliminary tests to induce inflammation. Based on these tests TNF-alpha was then chosen as most applicable inductor. The culture medium was replaced by corresponding serum-free medium containing TNF-alpha (5 ng/mL) 4 h before photosensitizer treatment was started.

IL-6 and IL-1beta Ready-Set-Go! ELISAs (eBioscience, Vienna, Austria) were performed according to the manufacturer's protocol to identify the best procedure for inducing a proinflammatory milieu, which was repeatedly checked throughout the study.

### 2.4. Uptake Experiments

After incubation with hypericin or ALA, respectively, for 1 h up to 8 h, cells were washed twice with 100 *μ*L DPBS and lyzed for 10 min by addition of 50 *μ*L 1% Triton X-100. Subsequently, the fluorescence intensity of the photosensitizers hypericin and PpIX in the cells was measured in 96-well plates (Greiner, Kremsmuenster, Austria) using a microplate reader (Infinite M200pro, Tecan, Groedig, Austria). Hypericin fluorescence was detected at lambda(ex) = 340 nm and lambda(em) = 604 nm and PpIX fluorescence at lambda(ex) = 435 nm and lambda(em) = 635 nm. The fluorescence signals were related to the protein content of each sample (BCA assay; Fisher Scientific, Vienna, Austria) to correct for variations in the cell mass. Measurements were performed in triplicates and series were repeated independently for at least two more times.

### 2.5. Data Analysis, Statistics

Comparisons between data points were statistically evaluated by the Student's *t*-test for independent samples. At least three independent series were included in the analysis.

## 3. Results

The results show the analysis of the fluorescence kinetics of EB and non-EB cell lines under normal and proinflammatory conditions. In order to check for the proinflammatory state of the selected cell lines after TNF-alpha induction, the IL-6 level of cells was checked at random (data not shown).

All cell lines take up hypericin or generate PpIX, respectively, at the selected concentrations.

**Figure 1:**
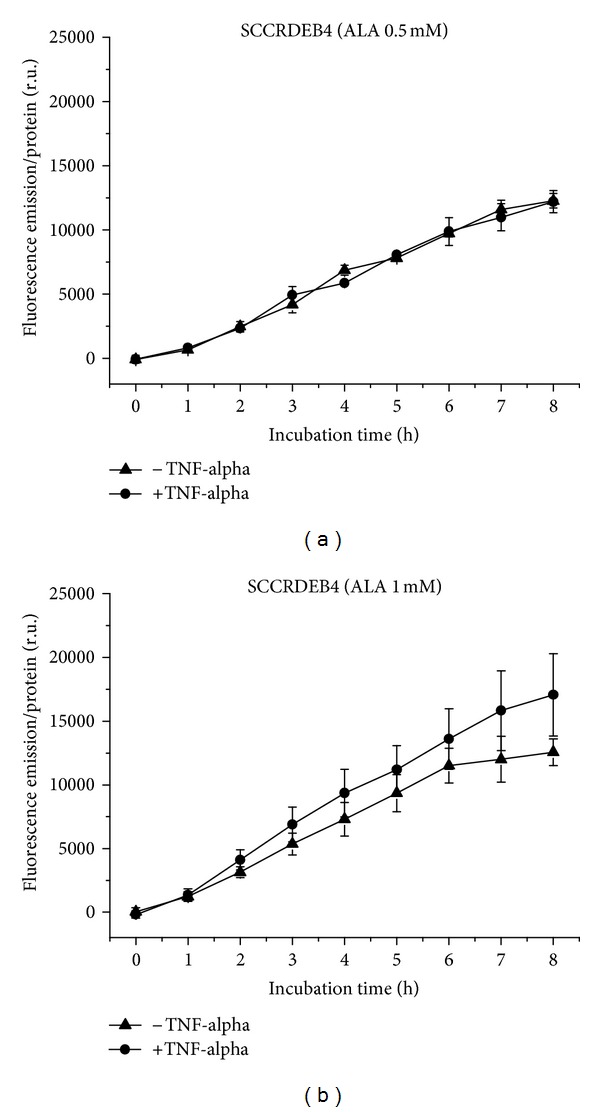
PpIX formation in SCCRDEB4 cells over 8 h, with and without TNF-alpha induction. (a) Application of 0.5 mM (b) of 1 mM ALA.

SCCRDEB4 cells generate PpIX from 0.5 mM ALA with linear kinetics, equally under normal and proinflammatory conditions (Figure 1(a)), and from 1 mM ALA with linear kinetics and moderately higher in the proinflammatory state (Figure 1(b)). However, these variations are not significant. This differs from normal state, which shows a final fluorescence intensity similar to the lower concentration but with a curve shape forming a plateau.

**Figure 2:**
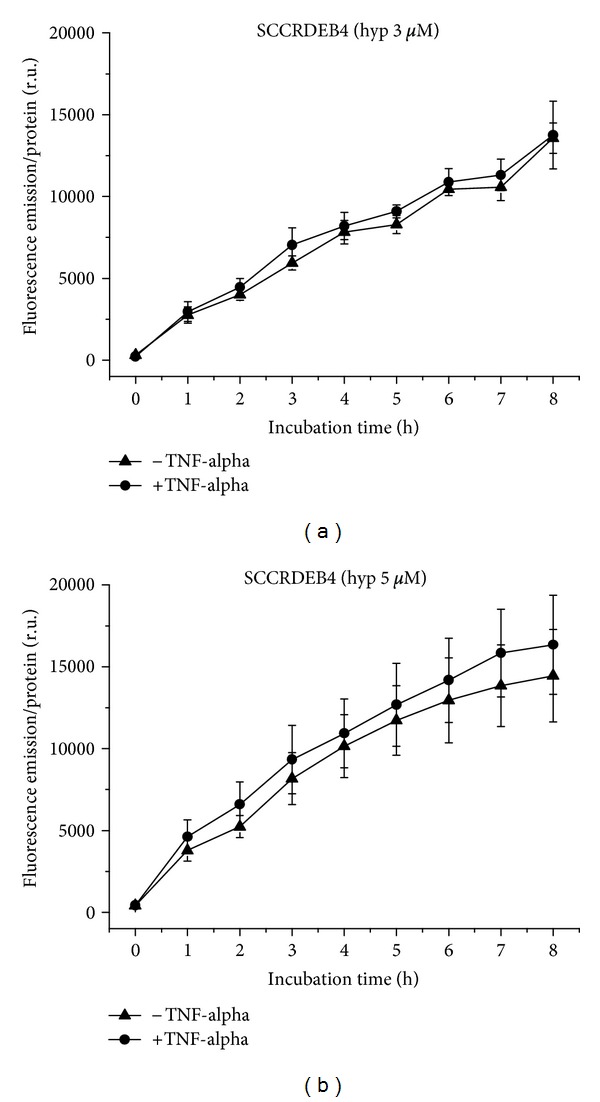
Hypericin uptake to SCCRDEB4 cells over 8 h, with and without TNF-alpha induction. (a) Application of 3 *μ*M, (b) of 5 *μ*M hypericin.

SCCRDEB4 cells take up hypericin at a concentration of 3 *μ*M with linear kinetics, equally under normal and proinflammatory conditions (Figure 2(a)), and at a concentration of 5 *μ*M almost equally under normal and proinflammatory conditions with curve shapes showing the onset of a plateau. The final fluorescence level at the proinflammatory state is marginally higher than the fluorescence after 3 *μ*M hypericin (Figure 2(b)).

**Figure 3:**
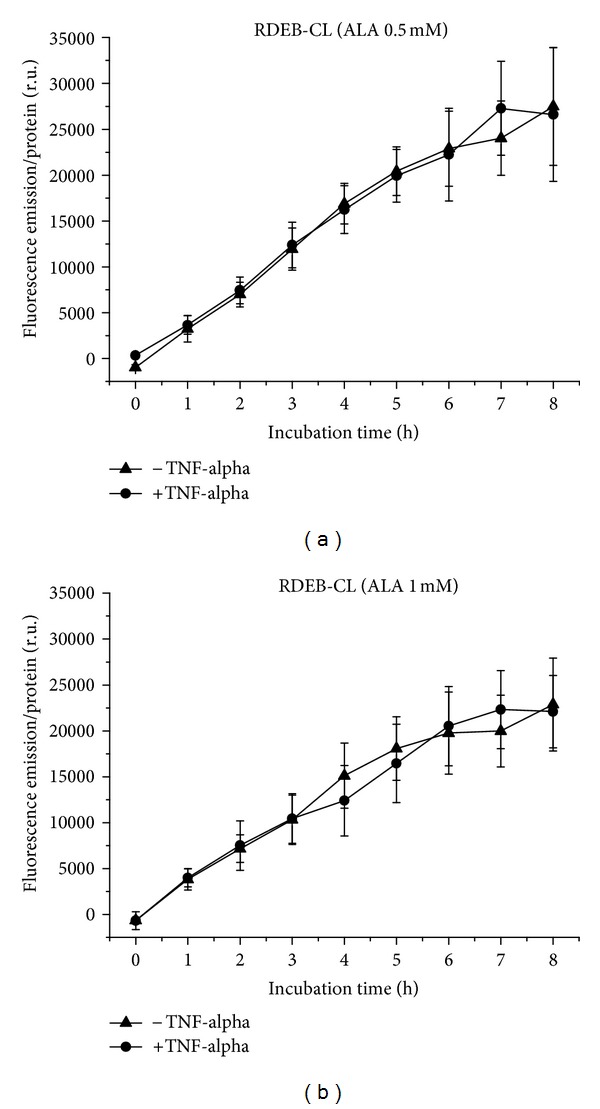
PpIX formation in RDEB-CL cells over 8 h, with and without TNF-alpha induction. (a) Application of 0.5 mM (b) of 1 mM ALA.

PpIX is generated by RDEB-CL cells from 0.5 (Figure 3(a)) and 1 mM ALA (Figure 3(b)) equally under normal and proinflammatory conditions, with more or less linear kinetics for 0.5 mM and with a beginning flattening to a plateau for 1 mM ALA. The fluorescence intensity is slightly lower at the higher concentration.

**Figure 4:**
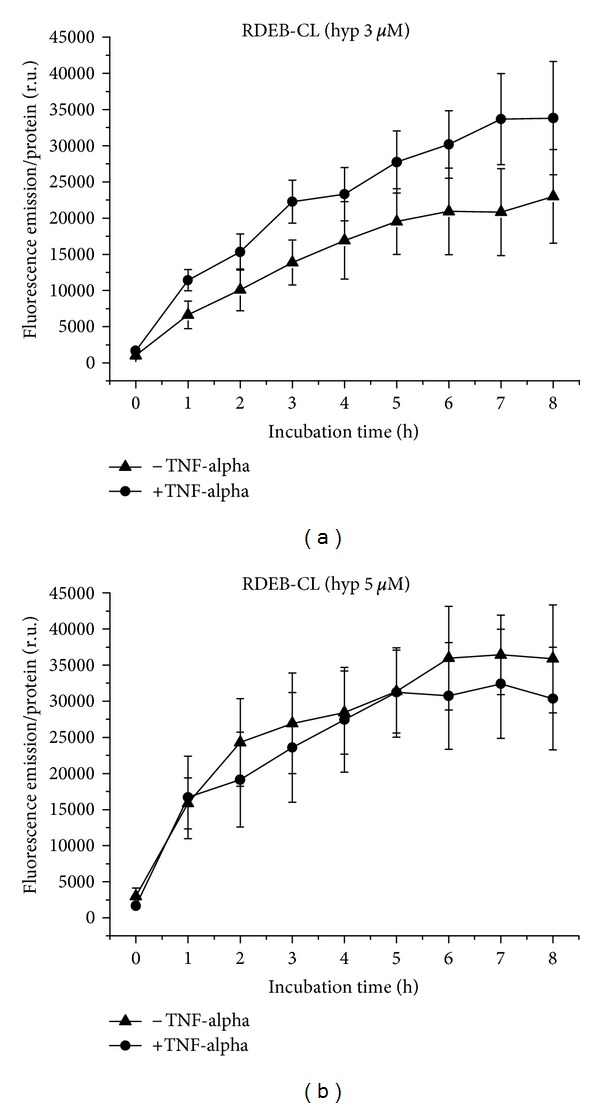
Hypericin uptake to RDEB-CL cells over 8 h, with and without TNF-alpha induction. (a) Application of 3 *μ*M, (b) of 5 *μ*M hypericin.

RDEB-CL cells take up hypericin at a concentration of 3 *μ*M in a moderately flattening curve course, statistically nonsignificantly higher under proinflammatory than normal conditions (Figure 4(a)). Uptake of 5 *μ*M hypericin occurs in a curve forming a plateau also more or less equally under normal and proinflammatory conditions (Figure 4(b)). Under normal conditions, the fluorescence increases with the hypericin concentration, but under TNF-alpha pretreatment, the final fluorescence values at 8 h incubation time with 3 and 5 *μ*M hypericin are similar (see also Figures 15(c) and 15(d)).

**Figure 5:**
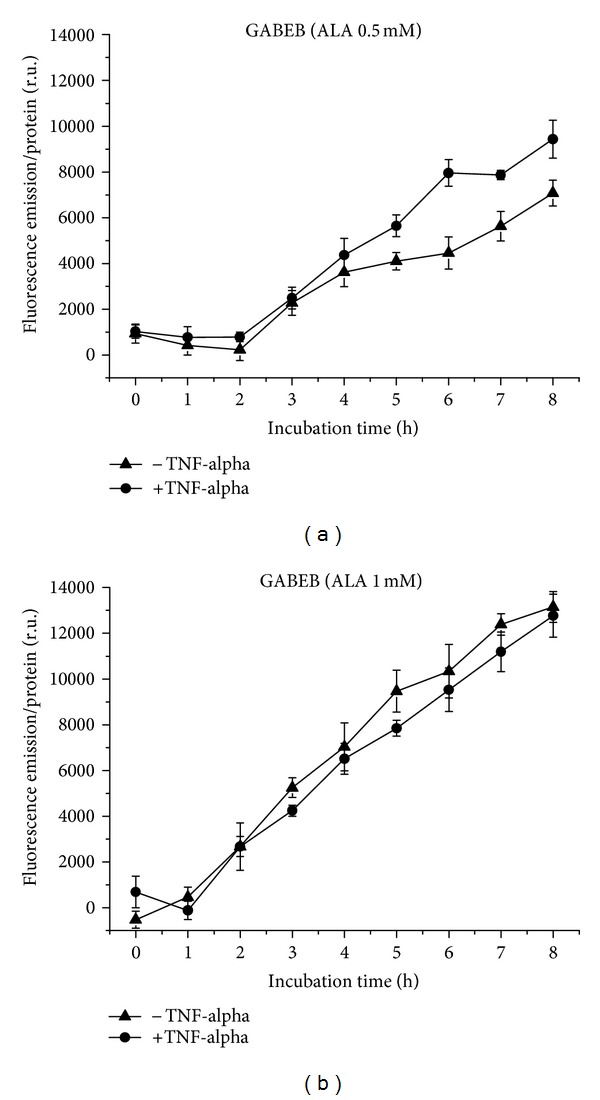
PpIX formation in GABEB cells over 8 h, with and without TNF-alpha induction. (a) Application of 0.5 mM (b) of 1 mM ALA.

GABEB cells generate PpIX from 0.5 mM ALA in a linear correlation with the incubation times, with a delay of 2 h. Under proinflammatory conditions, the fluorescence between 5–7 h is significantly increased (*P* ≤ 0.05, Figure 5(a)), in contrast to 1 mM ALA, which induces about equal PpIX fluorescence in a linear relation with a delay of 1 h (Figure 5(b)). Fluorescence intensity increases with ALA concentration and is about double without TNF-alpha induction.

**Figure 6:**
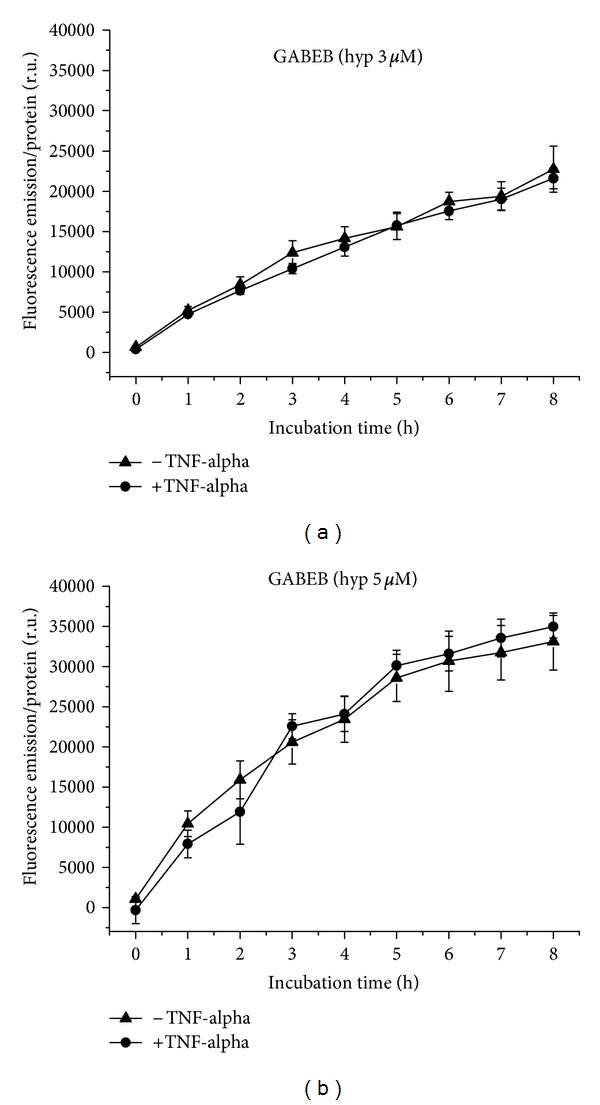
Hypericin uptake to GABEB cells over 8 h, with and without TNF-alpha induction. (a) Application of 3 *μ*M, (b) of 5 *μ*M hypericin.

Hypericin at a concentration of 3 *μ*M is taken up by GABEB cells in a linear curve course, equal under proinflammatory and normal conditions (Figure 6(a)). While the uptake of 5 *μ*M hypericin occurs also more or less equally under normal and proinflammatory conditions, the curve shape shows here a plateau (Figure 6(b)). Under both conditions, fluorescence increases with hypericin concentration.

**Figure 7:**
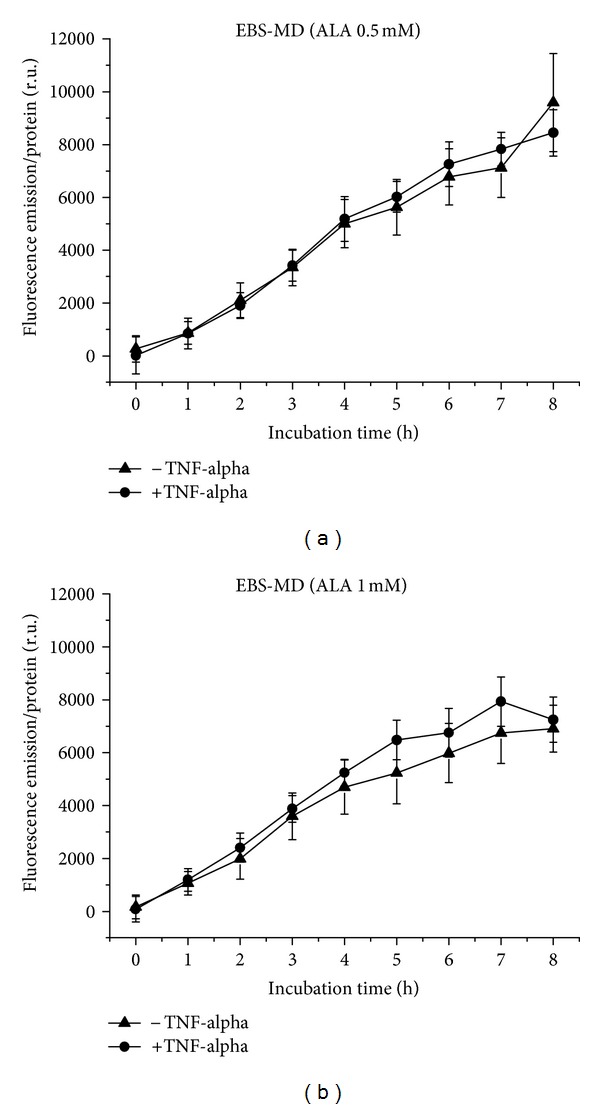
PpIX formation in EBS-MD cells over 8 h, with and without TNF-alpha induction. (a) Application of 0.5 mM (b) of 1 mM ALA.

EBS-MD cells generate PpIX from 0.5 mM ALA with linear kinetics up to 8 h (Figure 7(a)). The course is equal under proinflammatory and normal conditions, for 0.5 mM, and more or less also for 1 mM ALA (Figure 7(b)). However, PpIX formation after application of 1 mM ALA leads to a curve shape with a plateau. Noteworthy is the fact that the fluorescence decreases with increasing ALA concentration.

**Figure 8:**
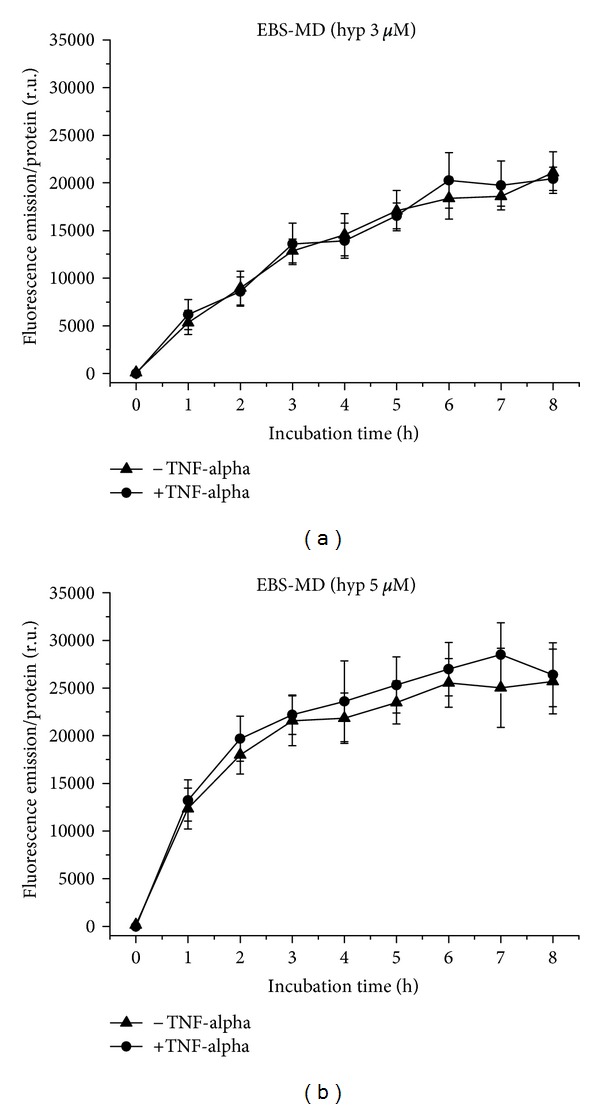
Hypericin uptake to EBS-MD cells over 8 h, with and without TNF-alpha induction. (a) Application of 3 *μ*M, (b) of 5 *μ*M hypericin.

EBS-MD cells take up hypericin at a concentration of 3 *μ*M in a moderately flattening curve course, equal under proinflammatory and normal conditions (Figure 8(a)). While the uptake of 5 *μ*M hypericin occurs also more or less equally under normal and proinflammatory conditions, the curve shape shows a distinct plateau and a very rapid increase already within the first two hours (Figure 8(b)). Under both conditions, fluorescence increases with hypericin concentration.

**Figure 9:**
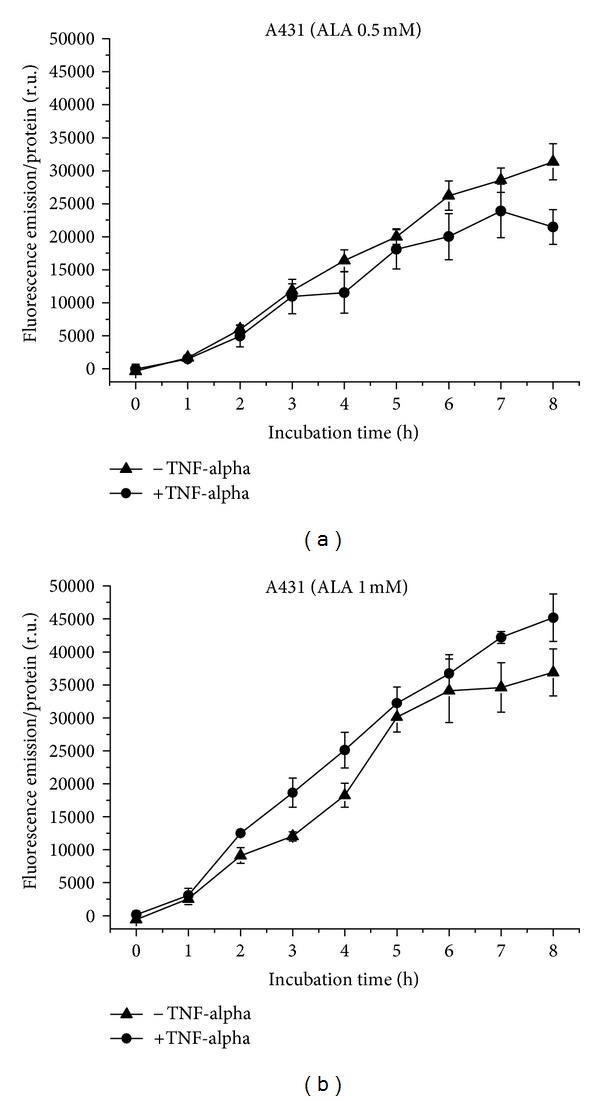
PpIX formation in A431 cells over 8 h, with and without TNF-alpha induction. (a) Application of 0.5 mM (b) of 1 mM ALA.

PpIX is generated by A431 cells from 0.5 mM ALA with linear kinetics up to 8 h under noninflammatory conditions (Figure 9(a)). Under proinflammatory conditions, the final fluorescence intensity is significantly (*P* ≤ 0.05) reduced, and the curve reaches a plateau. PpIX formation after application of 1 mM ALA shows a linear curve under proinflammatory conditions, which is flattened under normal conditions with lower fluorescence endpoints. A significant difference can be found for 2 and 3 h (*P* ≤ 0.05) (Figure 9(b)). Fluorescence increases with increasing ALA concentration.

**Figure 10:**
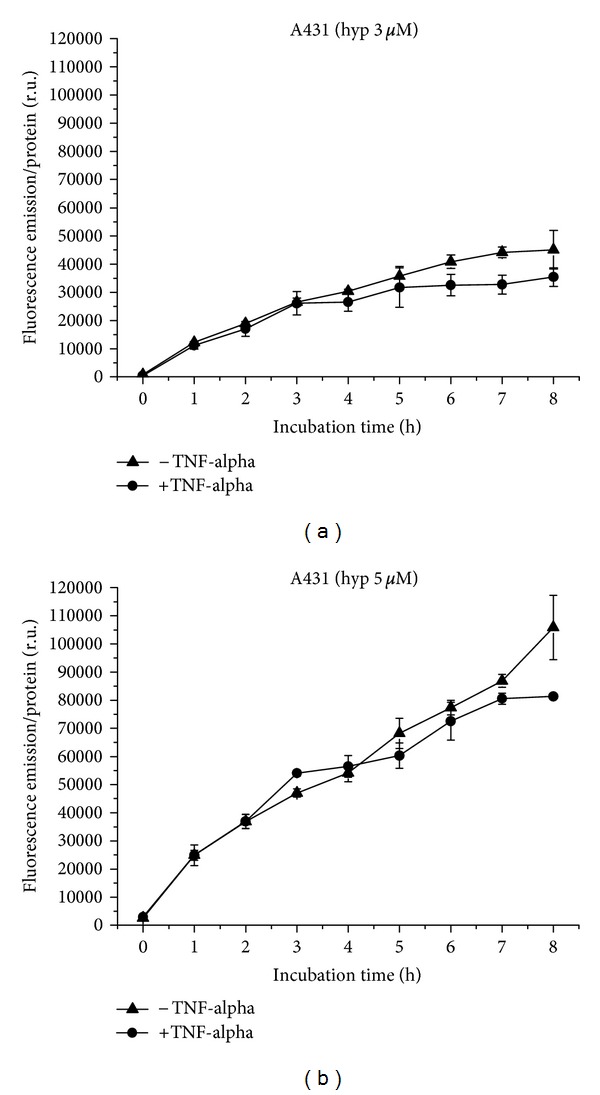
Hypericin uptake to A431 cells over 8 h, with and without TNF-alpha induction. (a) Application of 3 *μ*M, (b) of 5 *μ*M hypericin.

A431 cells take up hypericin at a concentration of 3 *μ*M in a moderately flattening curve course, equal under proinflammatory and normal conditions until 5 h. Between 5 and 8 h, the fluorescence increase is reduced under proinflammatory conditions with statistical significance at 7 h (*P* ≤ 0.05) (Figure 10(a)). Uptake of 5 *μ*M hypericin shows a similar curve course under proinflammatory and normal conditions, which is linear until 7 h. After 7 h, a reduced fluorescence increase after TNF-alpha application leads to a moderate difference in both conditions (Figure 10(b)). However, under both conditions, fluorescence increases more than doubles with increasing hypericin concentration.

**Figure 11:**
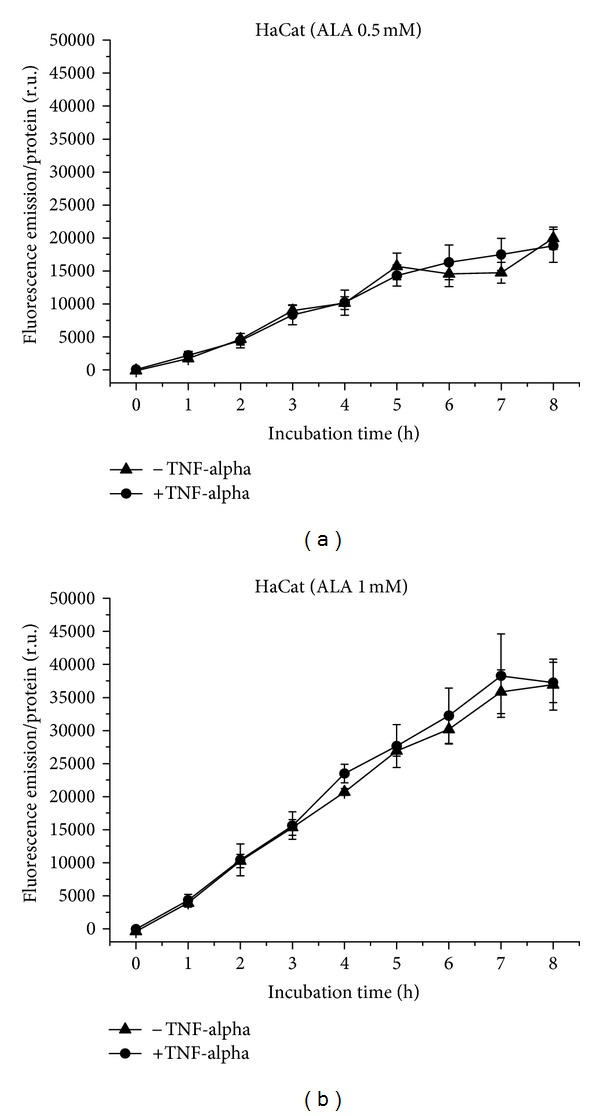
PpIX formation in HaCat cells over 8 h, with and without TNF-alpha induction. (a) Application of 0.5 mM (b) of 1 mM ALA.

HaCat cells generate PpIX from 0.5 (Figure 11(a)) and 1 mM ALA (Figure 11(b)) equally under normal and proinflammatory conditions with more or less linear kinetics. The only difference is that the fluorescence of PpIX induced by 1 mM ALA shows no further increase from 7 to 8 h. The fluorescence intensity shows almost double the amount with the higher concentration.

**Figure 12:**
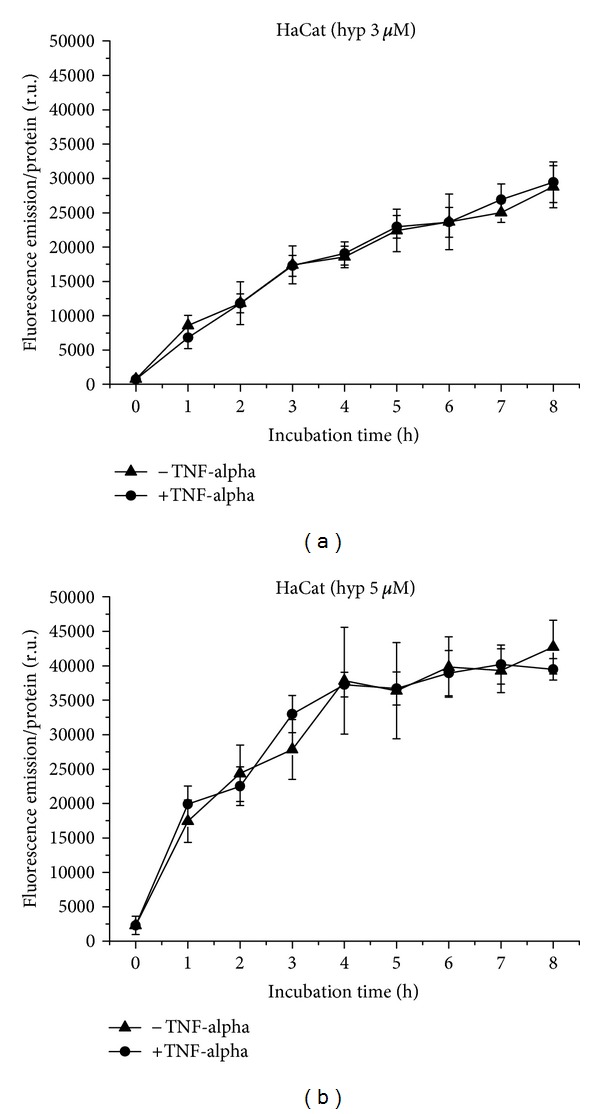
Hypericin uptake to HaCat cells over 8 h, with and without TNF-alpha induction. (a) Application of 3 *μ*M, (b) of 5 *μ*M hypericin.

Hypericin at a concentration of 3 *μ*M is taken up by HaCat cells in a moderately flattening curve shape, equal under proinflammatory and normal conditions (Figure 12(a)). While the uptake of 5 *μ*M hypericin occurs also more or less equally under normal and proinflammatory conditions, the curve shows a distinct plateau (Figure 12(b)) as well as a steep increase within the first hour. Under both conditions, fluorescence increases with hypericin concentration.

**Figure 13:**
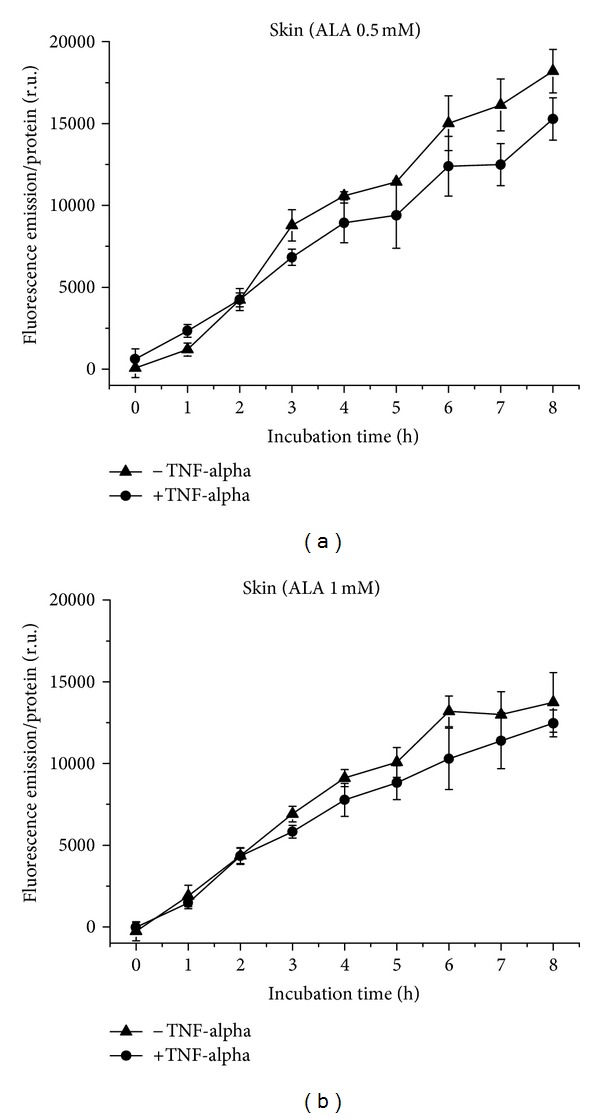
PpIX formation in Skin cells over 8 h, with and without TNF-alpha induction. (a) Application of 0.5 mM (b) of 1 mM ALA.

PpIX formation in Skin cells is about linear after incubation with 0.5 mM ALA (Figure 13(a)) and almost linear after incubation with 1 mM ALA (Figure 13(b)). Differences in fluorescence between inflammatory and normal conditions are not significant. Fluorescence decreases with increasing ALA concentration.

**Figure 14:**
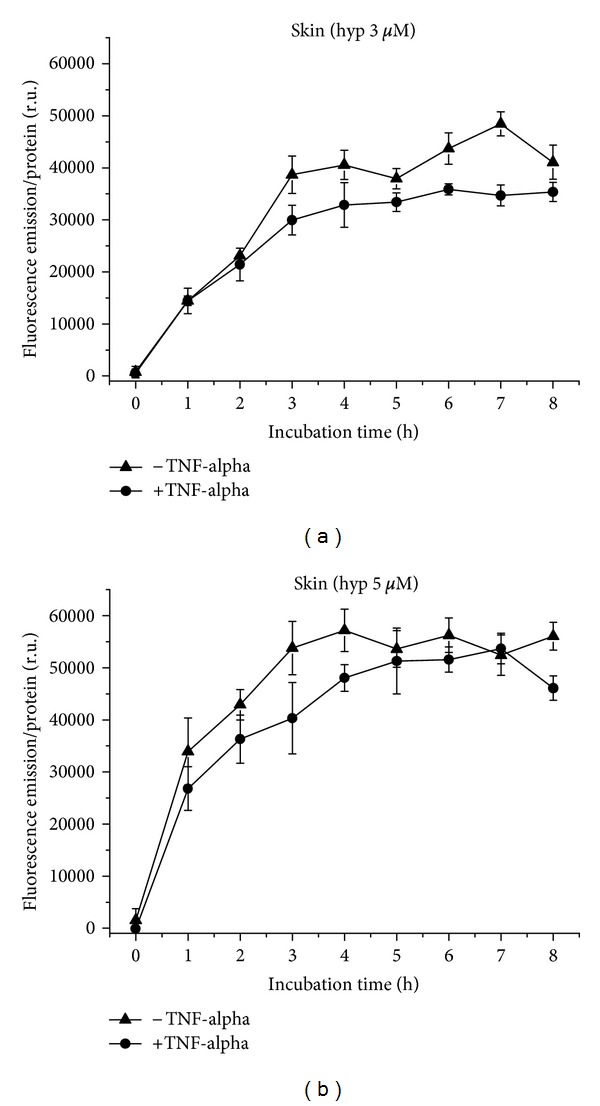
Hypericin uptake to Skin cells over 8 h, with and without TNF-alpha induction. (a) Application of 3 *μ*M, (b) of 5 *μ*M hypericin.

**Figure 15:**
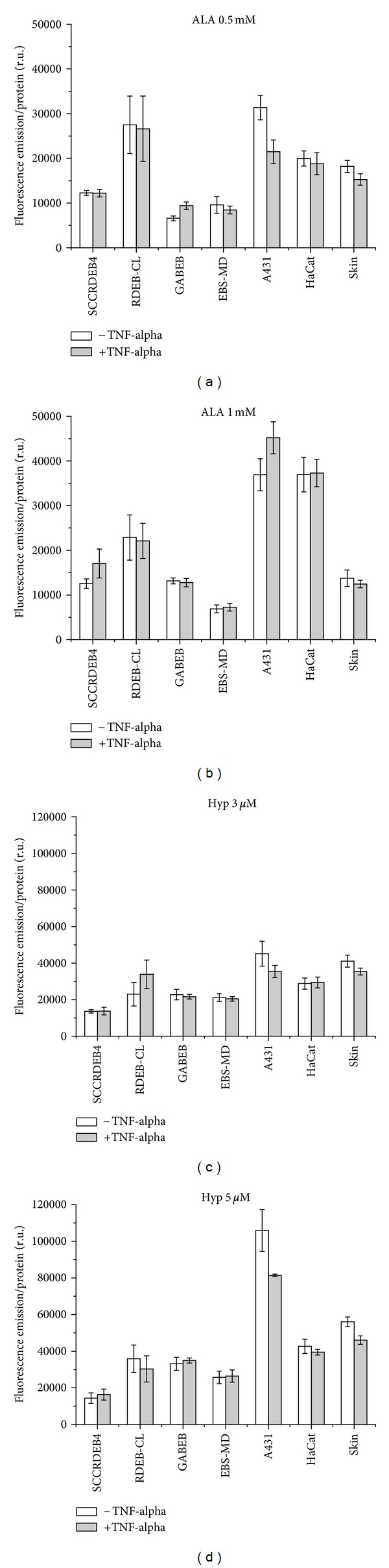
Relative photosensitizer fluorescence intensity in all cell lines at 8 h incubation time, with and without TNF-alpha. (a) Application of 0.5 mM ALA, (b) 1 mM ALA, (c) 3 *μ*M hypericin, and (d) 5 *μ*M hypericin.

Skin cells take up hypericin at concentrations of 3 *μ*M (Figure 14(a)) and 5 *μ*M (Figure 14(b)) in curve courses with plateaus under proinflammatory as well as normal conditions. Under proinflammatory conditions, the fluorescence of hypericin, 3 or 5 *μ*M, respectively, is lowered, highly significant for 6 and 7 h incubation with 3 *μ*M hypericin (*P* ≤ 0.01), and significant for the final value at 8 h (*P* ≤ 0.05) for 5 *μ*M hypericin. Under both conditions, fluorescence increases with hypericin concentration (see also Figures 15(c) and 15(d)).

## 4. Discussion

The aim of this study was the analysis of fluorescence kinetics of photosensitizers in EB cell lines representing the three types of EB, to assess the suitability of FD for squamous cell carcinoma of EB patients. Since proinflammatory conditions due to the chronic wounds [10] are often present in EB-patients, and excessive accumulation of photosensitizers in inflamed areas had been occasionally observed in the clinical situation, the fluorescence kinetics were also measured in all cell lines after TNF-alpha induction.

For this purpose, the photosensitizer hypericin and the precursor ALA were externally applied; the latter one to induce the endogenous generation of the photosensitizer PpIX in the cells. 

All cell lines produce PpIX and take up hypericin, as demonstrated by the fluorescence measurements, but with different accumulation kinetics. Since all cell lines represent fibroblasts or keratinocytes, this is in line with other studies [35–37].

Based either on the fluorescence kinetic curves or their endpoints at 8 h (Figures 15(a)–15(d)), the following comparisons can be made with special emphasis on the applicability of FD for SCC of EB-patients.

### 4.1. Curve Shape and Specific Accumulation of Each Photosensitizer in All Cell Lines

A moderate-curve flattening until to a distinct plateau is found in all cell lines after 5 *μ*M hypericin incubation and in most cases also after 3 *μ*M hypericin. In cell lines where it is found after ALA incubation, it is limited to 1 mM. 

Curves with a plateau normally indicate saturation with the photosensitizer but could in some cases be based on aggregation of the molecules, which quenches the fluorescence [38]. Especially the application of 5 *μ*M hypericin seems to be at the upper limit, and incubation with a higher concentration cannot be recommended for the used cell lines.

A retarded fluorescence increase was observed after ALA incubation in GABEB cells and a rapid initial increase after 5 *μ*M hypericin incubation of EBS-MD, HaCat, and Skin cells. The latter phenomenon points to a rapid initial uptake of hypericin mainly to fibroblast cell lines, which are known to carry out increased receptor-mediated endocytosis of low-density lipoprotein (LDL) in contrast to keratinocytes [39]. Lipophilic photosensitizers such as hypericin bind reportedly to LDL [40, 41]. However, uptake kinetics soon reach a distinct plateau in most cell lines, including Skin fibroblasts.

### 4.2. Effect of Concentration on the Photosensitizer Accumulation

In most cell lines, higher photosensitizer or precursor concentrations induce higher or—in the case of saturation—at least equal fluorescence (Figures 15(a)–15(d)). The observed seeming decrease in a few cell lines is not statistically significant.

### 4.3. Tumor Selectivity of EB Cell Lines (SCCRDEB4 versus RDEB-CL)

SCCRDEB4 cells show lower fluorescence levels than RDEB-CL cells, independent of TNF-alpha induction. This negative selectivity is the highest (more than double) after 0.5 mM ALA incubation, about double after hypericin treatment, with both concentrations, and reduced after 1 mM ALA (Figures 15(a)–15(d)). However, a statistically significant fluorescence reduction (*P* ≤ 0.05) was only detected with 5 *μ*M hypericin. In spite of its negative *in vitro* selectivity, hypericin tumor selectivity *in vivo* might well be existent [42].

RDEB-CL cell fluorescence is equal to or higher than that of the other EB cell lines.

### 4.4. EB-Specific Photosensitizer Accumulation in Malignant Cells (SCCRDEB4 versus A431)

The comparison of squamous cell carcinoma cells of an RDEB patient with those of a non-EB patient shows a 2-3 times higher fluorescence of A431 cells after PpIX formation and after 3 *μ*M hypericin accumulation, and an up to 8 times higher fluorescence after 5 *μ*M hypericin, for both pretreatment conditions (with and without TNF-alpha induction) (Figures 15(a)–15(d)). All differences are highly significant (*P* ≤ 0.01) with the only exception of a *P* ≤ 0.05 significance for 0.5 mM ALA in the proinflammatory state. The EB background of the malignant cells leads to drastically reduced fluorescence by decreased PpIX formation or hypericin uptake. Due to constant deficiency compensation processes of the recessive dystrophic EB cell line [43], other physiological activities could be limited.

### 4.5. Tumor Selectivity of Non-EB Cell Lines (A431 versus HaCat and Skin Cells)

The fluorescence of PpIX is significantly higher in A431 cells than in HaCat and Skin cells under noninflammatory conditions when incubated with 0.5 mM ALA (both: *P* ≤ 0.01) and significantly higher than in Skin cells under both conditions when incubated with 1 mM ALA (both: *P* ≤ 0.01). All three cell lines take up 3 *μ*M hypericin in a similar rate. When the cells are incubated with 5 *μ*M hypericin, the dye is accumulated to a much higher degree in A341 cells than in HaCat and Skin cells (highly significant with *P* ≤ 0.01), independent of TNF-alpha pre-treatment, (Figures 15(a)–15(d)). Except for 3 *μ*M hypericin and restricted for 1 mM ALA, the protocols are suitable to generate tumor selectivity in non-EB cell lines, at least under noninflammatory conditions.

### 4.6. EB-Specific Photosensitizer Accumulation in Non-Malignant Cells (GABEB versus HaCat and EBS-MD versus Skin)

Cells of junctional EB patients (GABEB), compared to keratinocytes (HaCat) as well as cells of an EB simplex patient compared to primary skin fibroblasts as their respective non-EB reference cell lines, show similar properties in the differential accumulation of photosensitizers (Figures 15(a)–15(d)): PpIX formation is significantly reduced in GABEB and EBS-MD cells for both conditions (all: *P* ≤ 0.01, except for GABEB, 0.5 mM ALA and TNF-alpha, and EBS-MD, 1 mM ALA without TNF-alpha: both: *P* ≤ 0.05). Hypericin uptake is also significantly reduced in GABEB cells when applied at a concentration of 3 *μ*M after TNF-alpha induction (*P* ≤ 0.05) and in EBS-MD fibroblasts under all treatments (all: *P* ≤ 0.01).

This confirms the evaluation made before concerning the comparison of the malignant cell lines (SCCRDEB4 versus A431 cells): EB cell lines seem to be physiologically less active resulting in reduced photosensitizer uptake or formation. Besides that, the underexpression of the low-density-lipoprotein receptor expression in the GABEB, EBS-MD and RDEB-CL cell lines, which was described by Knaup et al. [44], could also play a role—at least with regard to the decreased hypericin uptake.

### 4.7. Effect of TNF-Alpha-Induced Proinflammatory State on Photosensitizer Accumulation

In general, the proinflammatory state shows no major influence on photosensitizer accumulation as only a few nonsignificant differences to the noninflammatory state were found. Significantly increased fluorescence after TNF-alpha induction is restricted to the presence of PpIX in GABEB cells (5–7 h with 0.5 mM ALA) and A431 cells (2-3 h with 1 mM ALA). Reduced fluorescence was measured in A431 cells (incubated with 0.5 mM ALA and 3 *μ*M hypericin) and in Skin cells after hypericin application.

Concomitant measurements for checking the proinflammatory state of the cells showed that GABEB cells generate high levels of IL-6 and Skin cells even double the amount whereas A431 and HaCat cells present a low IL-6 level. Based on these data, it is hardly possible to correlate an influence of TNF-alpha induction on the fluorescence of the photosensitizers in the studied cell lines with the amount of released IL-6.

However, with our data, we support the hypothesis that the increased fluorescence found in inflamed tissue during FD of tumors is not due to higher accumulation of photosensitizers in nonimmune cells with a proinflammatory status but might rather be due to additional photosensitizer accumulation in the extracellular matrix and/or in infiltrating immune cells such as neutrophils, mast cells, monocytes, and macrophages [45, 46].

## 5. Conclusions

Following conclusions can be drawn from the results above.

1. All cell lines take up hypericin or generate PpIX mostly increasing with the incubation time, but with different kinetics.
2. SCCRDEB4 cells take up less hypericin and generate less PpIX than the nonmalignant RDEB-CL cells. This is in contrast to the non-EB cell lines, which show tumor selectivity.
3. From the here found *in vitro* results, we cannot conclude whether fluorescence diagnosis of SCC in EB patients will be feasible. Even though the applied photosensitizers exhibit no tumor selectivity *in vitro*, their tumor selectivity *in vivo* might well be existent.
4. EB cell lines are less active than non-EB cell lines concerning uptake of hypericin or formation of PpIX.
5. Since uptake of hypericin or formation of PpIX is hardly modified under proinflammatory conditions, the proinflammatory state of the cells seems to have no influence on the fluorescence detection of the photosensitizers. Therefore, the higher fluorescence of inflamed areas in tissue might rather be due to photosensitizer accumulation in infiltrating immune cells.

## Figures and Tables

**Table 1:** Cell lines, EB subtype, and mutations.

Cell line	EB Subtype	Mutation
RDEB-CL	RDEB-sev gen	COL7A1 (7786DG/R578X)
SCCRDEB4	RDEB-sev gen	COL7A1 (8244dupC/8244dupC)
GABEB	JEB-nH gen	COL17A1 (4003delTC/4003delTC)
EBS-MD	EBS-MD	PLEC1 (1287ins3/Q1518X)
HaCat	—	Wild type keratinocytes
Skin	—	Wild type fibroblasts
A431	—	epidermoid squamous cell carcinoma
